# Prehospital thrombolytic treatment of acute ischemic stroke using a remotely controlled CT scanner

**DOI:** 10.1038/s41598-025-19782-1

**Published:** 2025-10-14

**Authors:** Susanne Gaarden Ingebrigtsen, Linn Hofsøy Steffensen, Kurt Bøckman Gschib, Øyvind Solbakken, Tor Ingebrigtsen, Ellisiv Bøgeberg Mathiesen, Agnethe Eltoft

**Affiliations:** 1 Department of Neurology, University Hospital of North Norway, Tromsø, Norway; 2 Brain and Circulation Research Group, Department of Clinical Medicine, UiT The Arctic University of Norway, Tromsø, Norway; 3 Department of Radiology, University Hospital of North Norway, Tromsø, Norway; 4 Information and Technology Department, North Norway Health Trust, Tromsø, Norway; 5 Department of Neurosurgery, University Hospital of North Norway, Tromsø, Norway

**Keywords:** Health care, Medical research, Neurology

## Abstract

Timely access to diagnosis and treatment is crucial for improving stroke patients´ outcomes, but long prehospital transport times hinder timely treatment in rural areas. This study investigates the feasibility of using a remotely controlled computer tomography (CT) scanner at a decentralized medical center (DMC) to expedite prehospital intravenous thrombolysis (IVT) for acute ischemic stroke (AIS). The study involved three phases: technical implementation and testing, procedure development, and clinical training. We used a Siemens Healthineers Syngo Virtual Cockpit system to remotely control the CT scanner at the DMC. This enabled use of the scanner without a radiographer on call at the DMC. Eligibility criteria for undergoing prehospital IVT at the DMC were established. Paramedics, nurses, and physicians underwent comprehensive training on stroke assessment, CT scanner operation, and communication protocols. Technical testing demonstrated excellent feasibility of the system. Simulation exercises showed efficient teamwork, achievement of door-to-needle times (DNT) below 20 min, and NIHSS score consistency in 95% of cases. Risk assessment identified potential challenges, with mitigation strategies implemented. This study demonstrates the feasibility of leveraging existing infrastructure to remotely control a CT scanner at a DMC, for prehospital IVT in a rural setting. This approach is promising for improving timely stroke diagnosis and treatment in rural areas.

## Introduction

The annual incidence of stroke in Norway ranges from 10,000 to 11,000 cases, equating roughly to 200 strokes per 100,000 inhabitants per year^1^. This number is expected to rise due to an ageing population^2^. Early reperfusion therapy with intravenous thrombolysis (IVT) significantly improves patients´ functional outcome in acute ischemic strokes (AIS) (85–90% of all strokes). Swift diagnostics and treatment are essential as efficacy of IVT diminishes with treatment delays^3^. Therefore, minimizing the onset-to-treatment time (OTT) is crucial to improve outcomes.

Intra-hospital treatment times have improved through quality improvement programs^4,5^, but IVT remains underused^6^. In Norway, treatment rates vary from 7% to 29%^1^ with densely populated areas generally achieving higher rates compared to more rural regions^1,5,7^. This disparity is largely caused by prolonged transport time in rural areas such as Northern Norway, which often results in patients missing the critical 4.5-hour treatment window for IVT^1,5,8^.

To address this challenge, new prehospital solutions are needed to shorten onset-to-treatment (OTT) times. Mobile stroke units (MSUs) equipped with onboard CT scanners have been shown to reduce OTT and improve patient outcomes in urban settings^9–12^. However, their implementation is often unfeasible in rural areas due to long distances and low case volumes^13,14^. Alternative approaches tailored for such regions, including remote-controlled stationary CT scanners^15^, may thus be beneficial.

By allowing radiographers to remotely operate CT scanners, a system can be enabled for earlier diagnosis and treatment, reducing onset-to-treatment (OTT) times in AIS near the patient’s location without the need for resource-intensive on-site personnel. When combined with on-site paramedic assessments and real-time communication between local teams and stroke specialists, remote CT scanning can enhance IVT accessibility and streamline treatment workflows, potentially improving patient outcomes in regions with limited access to specialized stroke care.

We propose using remotely controlled stationary CT scanners in combination with on-site paramedic assessments and real-time communication with stroke specialists to enhance IVT availability and reduce OTT in rural regions. This study aims to evaluate the feasibility of this novel approach.

## Methods

### Setting

The University Hospital of North Norway (UNN) in Tromsø is a comprehensive stroke center (CSC) Serving Northern Norway, covering an area of approximately 113,000 square kilometers and a population of around 490,000 people. Additionally, it functions as the local hospital and stroke center for most of Troms county, with a population of approximately 130,000 and an area of 25,877 square kilometers. Furthermore, the hospital houses an Emergency Medical Communication Center and an air ambulance base. The District Medical Centre (DMC) at Finnsnes in Troms County is situated 170 kilometers by road from Tromsø (UNN Tromsø). The flight time by air ambulance is 40–60 min (excluded time from dispatch to take-off) and the transportation time by ground ambulance is 2.5-3 h.

The DMC provides emergency care for approximately 19,500 inhabitants across five rural municipalities within a 60-kilometer radius. During daytime, it offers outpatient and radiology services, while it is staffed out-of-hours by one primary care physicians and two nurses, with the ambulance service located in the adjacent building. The annual incidence of suspected stroke in the DMCs’ catchment area is estimated to be around 40–50 cases (0.2–0.25%)^1^. Based on nationwide stroke registry data^1^, approximately 20% of AIS patients receive IVT, reflecting those who arrive within the treatment window and are deemed eligible after clinical assessment^5^. Applying this proportion to the estimated annual incidence of strokes in the DMC catchment area (40–50 cases, 0.2–0.25%) suggests that around ten AIS patients should receive IVT annually in the region. This figure represents an estimate of expected treatment numbers rather than a formal eligibility rate and should therefore be interpreted with caution when planning service provision. Some degree of over-triage is necessary to avoid missing eligible patients, and stroke mimics constitute a substantial proportion of suspected acute strokes, with reported rates ranging from 20 to 50% depending on whether initial evaluation is performed by paramedics or stroke physicians^16,17^. Some patients per year may thus be assessed at the DMC and subsequently transferred to the CSC for further workup but ultimately receive a non-stroke diagnosis. Such transfers are often necessary to rule out stroke but may tie up resources that could be utilized more effectively. A CT scanner (SOMATOM Definition CT AS, Siemens Healthcare GmbH, Germany) for scheduled outpatient service is located at the DMC. The CT scanner is operated 3 days a week between 08.00 and 16.00 by radiographers but not readily available for emergency services.

### Overview

The study was structured in three phases: technical implementation, procedure development and clinical training. The technical implementation included testing and was conducted in parallel with the development of clinical procedures and training of the staff. A comprehensive assessment of technical and clinical/operational risks was conducted. To mitigate these risks, several measures were developed and implemented. Redundancy planning included backup power supplies and standardized restart procedures to ensure uninterrupted system operation during power outages. Security planning covered protocols for switching to alternative communication channels in the event of system failures, as well as procedures for managing CT scanner downtime, including direct patient transfer to the CSC. Additional measures involved continuous system monitoring, simulation-based training, regular drills, and ongoing education to reinforce staff competencies and maintain system reliability and preparedness.

### Technical implementation

#### Remotely controlled CT scan

In June to October 2021, we established the technical infrastructure enabling remote control of the CT system in collaboration with the IT department and the software manufacturer. We used Syngo Virtual Cockpit^18^, a multi-vendor remote scanning software developed by Siemens Healthineers. It securely connects to CT, MRI, and other imaging devices that support Syngo Expert-I. Expert-I is a software that enables radiographers to remotely access and control any CT, MRI, or other imaging modality connected via an Expert-I enabled keyboard, video, and mouse (KVM) switch. Prerequisites for the software is internet connection to the clinical network, Digital Imaging and Communications in Medicine (DICOM) standard compliance, adherence to minimal hardware specifications and adherence to data protection regulations (GDPR).

**Fig. 1:**
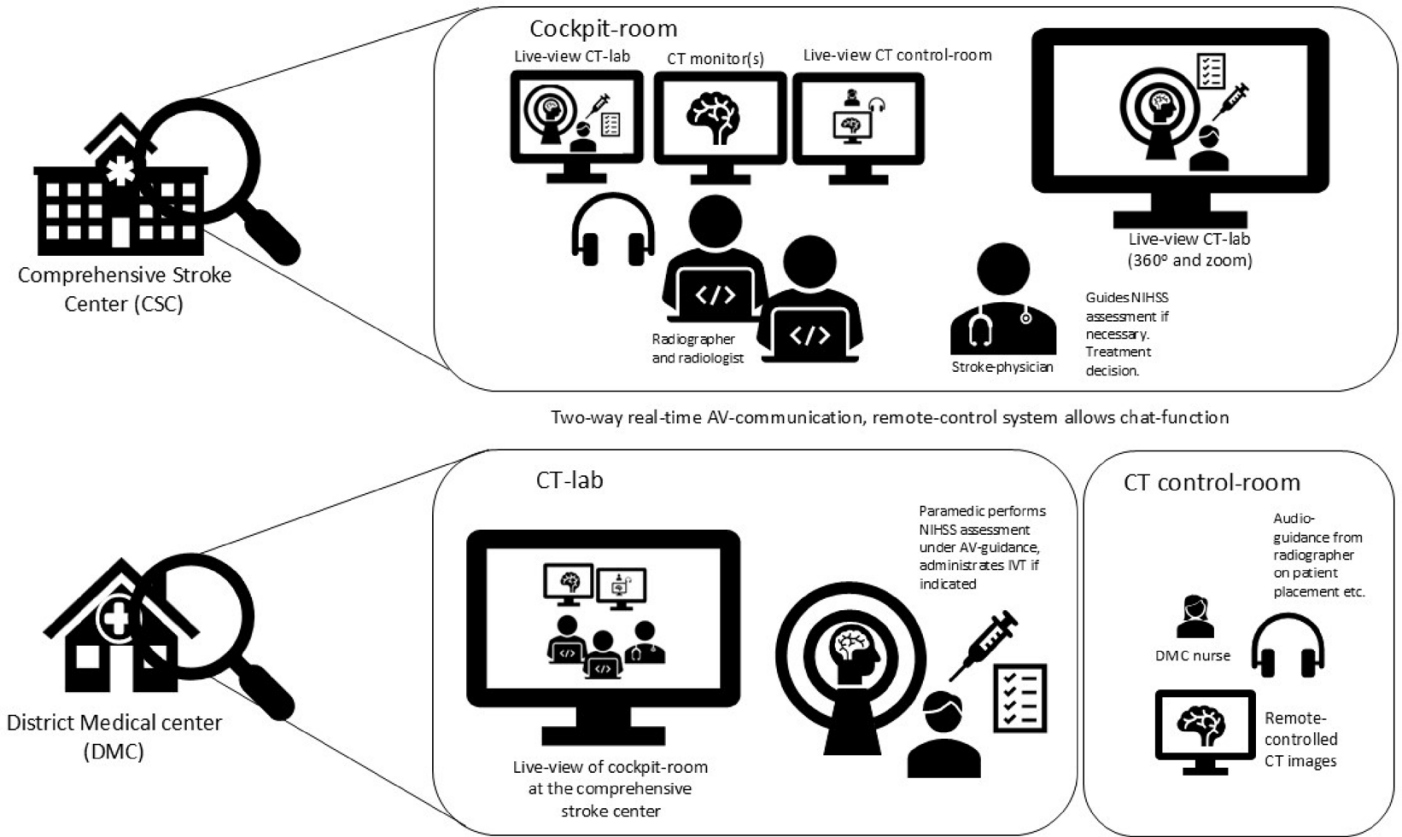
Set up for prehospital stroke treatment at the District Medical Center. Illustration of the setup for the cockpit solution at the comprehensive stroke center (CSC) and the CT lab at the District Medical Center (DMC). At the CSC, the stroke team is alerted, and the radiologist gains remote access to the cockpit solution, enabling control of the CT scanner located at the DMC. At the DMC, the local medical staff prepare the patient for scanning, connect monitoring equipment, and support the scanning procedure under remote guidance. NIHSS is performed by paramedics under AV-guidance from the stroke-physician at the CSC. Images acquired at the DMC are transmitted directly to the CSC for rapid assessment, allowing timely decision-making regarding thrombolytic treatment.

The cockpit system incorporates integrated audio and chat functions, with video provided by a third-party vendor. We installed two smaller video cameras in conjunction with the remotely controlled system. Camera 1 was mounted in the control room, allowing the radiographer at the CSC to monitor the computer screens that the nurse at the DMC is viewing. Camera 2 was positioned above the window from the control room to the CT scanner, replicating the perspective the radiographer would have had if present at the DMC. Figure 1 illustrates the cockpit solution at the CSC and the CT at the DMC.

The audio/video system used for clinical assessment and communication between paramedics and the junior stroke physician on call at the CSC featured a larger camera with zoom capabilities and 360-degree rotation. This camera was also employed to ensure correct patient positioning at the CT scanner.

We established a web-based service which provided staff access to educational content including demonstration videos and instructions on how to remotely control the CT scanner. Thirty-four radiographers were then trained one-by-one by the head radiographer at UNN. Procedures for admission and management of patients at the DMC were developed. Approximately 30 nurses at the DMC received one-to-one training in operating the CT scanner. This included system warm-up and patient positioning, all guided via audio/video guidance from the radiographer at the CSC both through the cockpit system and assisted by the audio-/video system for clinical assessment. Both training mannequins (training phantoms) and cabbage heads were used during this phase. Due to their ready availability, lighter weight, and ease of handling, cabbage heads were preferentially used for training in CT scanner positioning and parameter settings.

#### Technical testing

Image quality was established prior to the study through nearly 10 years of regular operation by radiographers and was equivalent to that at the CSC, although not previously tested in emergency settings. The remotely controlled CT scanning underwent rigorous testing procedures. Initial tests were conducted on practice mannequins (training phantoms). These were followed by trials on elective patients during daytime operations, with a radiographer present at the DMC and real-time audio/video guidance from the radiographer at the CSC.

Image quality and time-efficiency of remotely controlled and in-hospital acquisition was assessed by both radiographers and radiologists at the CSC, and any technical failures were documented and addressed to ensure optimal system performance.

##### Clinical procedure development

**Fig. 2:**
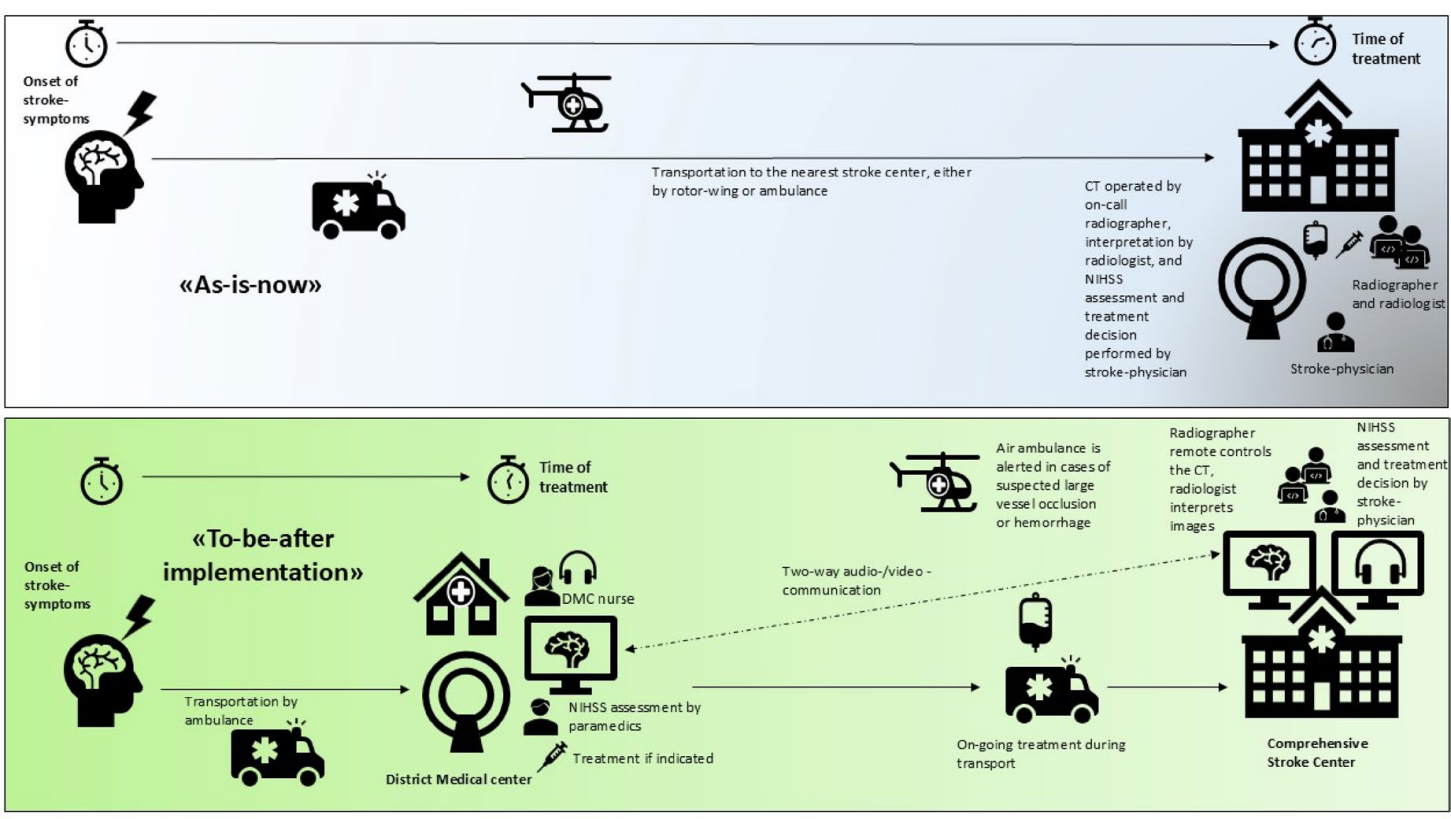
As-is-now, as well as to-be after-implementation. Illustration of the current situation (“as-is”) compared with the envisioned workflow after implementation (“to-be”). The “as-is” pathway depicts conventional patient transfer and delayed access to advanced imaging and treatment. The “to-be” scenario illustrates the streamlined process with remote CT access and earlier stroke team involvement, enabling faster diagnostics and treatment initiation.

A standardized operating procedure (SOP) for patient selection, transportation, and administration of IVT at the DMC was collaboratively developed by representatives from the ambulance service, DMC nurses, radiographers, radiologists, and stroke physicians at the CSC. The SOP outlines how the Emergency Medical Communication (EMC) center handles calls from patients or their caregivers reporting potential stroke symptoms. When a call is received via the local emergency number 113, the EMC dispatcher assesses the situation to determine if the symptoms align with a suspected stroke. If so, the dispatcher notifies both the local ambulance service and the on-call stroke physician at the CSC. The stroke physician then evaluates the patient’s eligibility for a stroke alert, which can be issued either at the CSC or the DMC. Figure 2 illustrates the situation as-is, as well as to-be after-implementation.

Eligibility criteria for stroke-alert at the DMC include:

- A clinical diagnosis of suspected stroke due to sudden onset of limb weakness, facial asymmetry, trouble walking, speaking, and/or vision disturbance.
- An anticipated time gain of at least 20 min if transported to the DMC instead of the CSC.
- Potential for IVT within 4.5 h of symptom onset.
- Absence of contraindications for IVT.

Patients with a possible AIS who do not meet these criteria are transported to the CSC for standard management according to national guidelines. The decision is made jointly by the dispatcher at the EMC and the on-call stroke physician at the CSC.

Paramedics collect medical history and perform an initial assessment using the ABCDEs framework for trauma care screening. If the patient is stable, they proceed with a National Institutes of Health Stroke Scale (NIHSS) assessment. Two peripheral intravenous lines are secured during transport by ground ambulance to the DMC.

DMC nurses are notified by the EMC of a suspect stroke. At the CSC, a stroke-alarm alerts the on-call stroke team (radiographer, radiologist, and junior stroke-physician) at least 10 min before expected patient-arrival at the DMC.

Paramedics then provide the junior stroke-physician with the medical history, including time of symptom onset and any known contraindications for IVT. NIHSS scoring is repeated if deemed necessary, overseen by the junior stroke-physician during real-time audio/video communication.

The nurse positions the patient in the scanner under real-time audio/video guidance by the radiographer, who remotely control the CT scanner and perform a non-contrast scan of the head via the cockpit solution. DMC nurses also provide a point-of-care laboratory blood test upon DMC arrival.

The radiologist at UNN interprets the images, treatment decision is made by the junior stroke-physician in conference with a stroke-specialist. If indicated, IVT (Alteplase) is administered by the DMC nurse according to the established protocol, following the standard dosage of 0.9 mg/kg body weight (maximum 90 mg). 10% of the total dose is given as an initial intravenous bolus over 1 min, followed by continuous infusion of the remaining 90% over 60 min. In prehospital settings, the infusion may be adapted to a controlled drip rate without a pump, while maintaining the same dosing guidelines, in line with the CSC protocols. The patient is closely monitored and transferred to the CSC by ground or air ambulance, as determined by the junior stroke physician. We chose not to implement contrast-enhanced CT in this study, aiming to minimize risks such as contrast extravasation, allergic reactions, contrast-induced nephropathy, and increased radiation exposure, although these complications are generally rare. If a large vessel occlusion (LVO) is suspected, based on a clinical NIHSS score of 8 or higher^19,20^, or the presence of a hyperdense artery sign on CT imaging^21^ the patient is prioritized for rapid transport by air ambulance to the CSC for assessment and potential endovascular treatment. If a patient undergoing IVT deteriorates or an intracranial haemorrhage due to IVT is suspected, a new CT is conducted immediately. Treatment for a haemorrhagic complication is then initiated on-site before transferral to the CSC by air ambulance for neurosurgical assessment. The DMC staff, including nurses and paramedics, are trained to manage acute complications like angioedema and respiratory or cardiac failure.

### Clinical training

#### Training programme

A total of 50 paramedics and 30 nurses at the DMC participated in a structured educational programme. This included a two-day on-site course taught by a paramedic, a radiologist and a stroke-physician who focused on the diagnostic assessment and treatment of acute stroke and incorporated practical exercises in NIHSS scoring. Completion of the course and obtaining online certification in NIHSS scoring was mandatory. All 12-junior stroke-physicians at the CSC were certified prior to the study, as integrated training for all new physicians.

#### Training evaluation

The program concluded with a simulation exercise involving a stroke alert scenario. Teams consisting of 1–2 nurses (DMC), 2–3 paramedics (DMC), 1 junior stroke-physician (CSC), 1 radiographer (CSC) and 1 radiologist (CSC) completed the simulations twice as a minimum.

The simulated cases primarily involved patients who met the inclusion criteria for transport to the DMC, resulting in a predominance of mild strokes (NIHSS < 8). Common stroke mimics such as epilepsy and infections were included, alongside some cases of intracerebral hemorrhage (ICH). The simulated cases did not include adverse events or treatment-related complications.

The evaluation used key performance metrics; door-to-needle time (DNT) and the degree of alignment between the assessed NIHSS score and the predefined NIHSS score for each scenario, which served as the gold standard.

##### Risk assessment

We used the International Organization for Standardization´s (ISO) guidelines^22,23^ to do a semiquantitative assessment to identify risks and possible adverse events (AE). The process involved the following steps:

- Identification of Risk Sources: Recognizing potential sources of technical and clinical risks.
- Analysis of Potential Occurrences and Their Likelihood: Assessing the likelihood and potential impact of identified risks.
- Determination of Preventive Measures: Establishing measures to prevent or mitigate these risks.
- Implementation of Actions and Follow-up Tasks: Executing the preventive measures and monitoring their effectiveness.

We scored probability based on likelihood and then ranged consequences accordingly (Table 1). Probability was scored as 1: low (likely to occur in less than 1/100 cases, < 1%), 2: moderate (likely to occur in more than 1/100 cases, ≥ 1%), 3: high (likely to occur in more than 1/20 cases, ≥ 5%); and 4: very high (likely to occur in more than 1/10 cases, ≥ 10%). Consequences were then ranged as 1: low; 2: moderate; 3: high; or 4: very high^22,23^.

## Results

### Technical testing

The quality of images acquired at the CT scanner at the DMC was thoroughly evaluated prior to startup by both radiographers and radiologists at the CSC. Additionally, no delays in image acquisition were observed, and the time efficiency of the remotely controlled acquisitions was deemed equivalent to in-hospital procedures, based on the experiences and observations of the operators and clinicians during the implementation phase.

There were no delays in the usage of audio, mouse, or keyboard in the cockpit solution. However, initially we experienced a 1–2 s delay using the third-party cameras. This issue was addressed early in the implementation phase, and the software provider updated the system, eliminating any delays.

The video and audio system, used for both patient positioning and clinical assessment, showed no delay. It supports a 360-degree view and zoom functionality, ensuring precise assessment and patient positioning.

Due to firewall restrictions between the DMC and the CSC, Bluetooth headsets were rendered unusable and had to be replaced by standard wired headsets. During early testing, it was discovered that if the nurse at the DMC attempted to log into the cockpit solution while the CT scanner was still warming up, the scanner would require a manual restart. This issue was addressed by clearly defining and communicating the precise sequence of operational steps that the nurse should follow, which was then emphasized during staff training and incorporated into the formal procedure. This clarification ensured smooth system startup and prevented the need for manual restarts.

An unforeseen challenge arose for radiographers during patient positioning due to differences in the patient tables at the DMC and the CSC. The DMC’s CT scanner table had a combined elevation and sliding mechanism, while the CSC’s CT scanner tables had separate functions for these movements. This was successfully addressed through one-on-one training sessions for all nurses and radiographers. To maintain competence, biannual full-day training sessions are held.

All technical issues were documented and resolved to ensure optimal system performance.

### Clinical training

Twenty-two teams (nurses, paramedics, neurologist, radiographer) participated in the simulations with two repetitions per drill. Paramedics and radiologists participated at least once. DMC nurses and junior stroke-physicians participated on average in 2 teams. All DNTs were under 20 min (range 9–19). Teams demonstrated a significant improvement from Round 1 (mean 15 min, range 11–19) to Round 2 (mean 11 min, range 9–14). The mean reduction in DNT was 4.0 min (95% CI 2.9–5.6, *p* < .0001). The paramedics´ NIHSS scores were consistent with the predefined scores in the scenarios in 42/44 (95%) sessions.

### Risk assessment

**Fig. 3:**
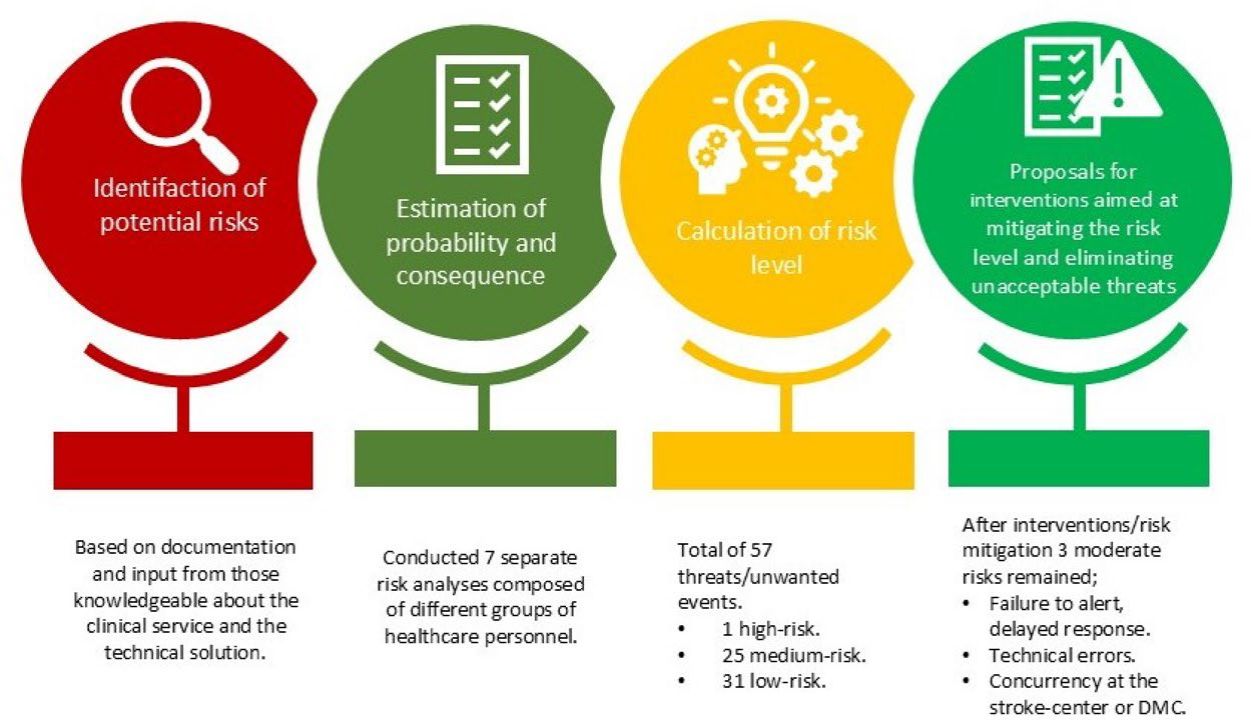
Risk assessment analysis. The different risk analysis groups had participants from the ambulance service, emergency medical central, nurses and on-call physicians at the District Medical Centre, neurologists, radiographers and radiologists from the comprehensive stroke center, representatives from the Medical Technology Department and Technical Department (video section), representatives from the IT Department, and Siemens.

A risk assessment was performed to identify potential risks and suggest mitigation strategies (Fig. 3). The risk-assessment identified 57 possible risks and AES. Only one risk was considered as very high (likely to occur at a rate of 10% of cases). A simplified table (Table 1) outlines the key risks and mitigation strategies within our approach. Potential risks included delays in treatment due to factors like human error, resource constraints, and communication issues. Mitigation strategies focus on improving protocols, staff training, and coordinating responses to ensure timely and effective treatment. For instance, the use of an app-based NIHSS and standardized checklists reduce errors in patient assessment and treatment. We also implemented an alert system for emergency personnel to minimize delays when responding to suspected stroke cases. After risk assessment and the implementation of mitigation strategies, three threats remained at moderate, indicated in yellow in the table (likely to occur ≥ 1%), while the remaining 32 were considered low, indicated in green in the table (likely to occur < 1%).

## Discussion

This study demonstrates the feasibility of using a stationary, remotely controlled CT scanner in conjunction with on-site paramedic evaluations and real-time audio/video communication with hospital-based stroke physicians for prehospital IVT in AIS.

In the simulated scenarios, all participating teams achieved DNTs well within the national quality goal of DNT within 40 min^1^ and demonstrated a significant improvement between successive drills. Furthermore, paramedics’ NIHSS scoring were highly consistent with the predefined scores, indicating reliable clinical assessments. By enabling CT imaging to be performed nearer to patient, this system has significant potential to reduce the time to diagnosis and initiation of IVT in rural areas.

Early intervention with IVT can lead to better patient outcomes, including reduced disability and mortality. This approach can expand access to specialized stroke care, particularly in rural or remote areas where timely transportation to a hospital with CT scanning capabilities may be challenging.

One of the primary challenges in extending prehospital care to stroke patients is differentiating between ischemic stroke and haemorrhagic stroke. Although emergency medical services (EMS) systems have developed protocols for rapid transportation to hospitals with CT scanners, delays in obtaining imaging and initiating treatment can significantly impact patient outcomes. To address this challenge, technological advancements have focused on bringing diagnostics, including CT scans for stroke patients, closer to the patient^9,24–27^, eliminating the need for transportation to a hospital for imaging. Telemedicine technologies can easily facilitate real-time communication between prehospital providers and stroke specialists, accelerating the decision-making process^28^.

An approach to reduce the OTT for IVT is the MSUs, equipped with CT scanners and specialized personnel. However, the implementation costs of MSUs vary significantly between countries and typically require a high patient volume to be cost-effective^14,29,30^. Another innovative approach involves integrating CT technology into helicopters for acute stroke treatment, particularly in rural areas. Helicopter-based CT units, while promising, come with high costs, requiring significant investment in specialized equipment, maintenance, training, and coordination, alongside safety considerations regarding radiation exposure in a confined mobile environment^25^.

A similar approach as in our study has also inspired the use of stationary CT in a rural area with on-call personnel. In Hallingdal, Norway, paramedics are trained to operate the local CT scanner at a DMC, and can initiate IVT in consultation with a hospital^31,32^. The results from this study indicate a possible reduction in time to IVT compared to traditional methods involving transport and in-hospital assessment.

Christensen et al. has conducted a hypothetical simulation-based pilot study comparing MSUs, the prehospital CT in Hallingdal, and helicopter-based CT. Their findings supporti prehospital treatment as a way to reduce onset-to-treatment time in AIS^33^. However, the optimal strategy depends on both geography and weather conditions.

Our approach builds upon these models, utilizing existing infrastructure with a novel, shelf-ready, remotely controlled CT scanner. The remotely controlled system functions with no time delay and delivers imaging quality equivalent to in-hospital scanners. A thorough risk assessment was performed, highlighting the importance of a coordinated and efficient approach to stroke care, involving paramedics, EMC, nurses at the DMC, junior stroke-physicians, radiographers and radiologists at the CSC.

Continuous sustainability is ensured through integrating training for new personnel into the basic paramedic education within our health region. This is supplemented by regular simulation sessions and biannual educational updates. New nurses at the DMC also receive training in CT scanner operation as part of their orientation. System performance is maintained through daily tests and established procedures for rebooting the system in case of power or connectivity issues. We have established a clear plan to implement continuous surveillance using Statistical Process Control (SPC)^34^ as part of an ongoing quality improvement initiative at the CSC, ensuring guideline adherence and actively monitoring the quality of both IVT services and simulation trainings at the CSC and DMC. SPC tracks processes over time, distinguishing normal variation from meaningful deviations in DNT, that require intervention.

Regarding the potential expansion of this approach, Norway currently has over 50 DMCs, primarily in rural areas. While not all centers have CT scanners, similar DMCs with remotely controlled CT could be established in other rural or semi-rural regions with limited access to advanced stroke care. This model brings imaging and treatment closer to patients, particularly in locations where mobile stroke units (MSUs) or full stroke centers are unfeasible. Further development to include remote-controlled contrast-enhanced CT could enable rapid detection of patients with a LVO, allowing direct routing to thrombectomy centers. Transition from alteplase to tenecteplase would further simplify IVT administration, by replacing a bolus-plus-infusion regimen with a single-dose injection. Together, these improvements could shorten time to reperfusion in AIS and ultimately improve functional outcomes while reducing long-term disability and healthcare burden.

The main costs involved in expanding this method include purchasing CT scanners, establishing reliable telecommunication systems, and training paramedics, nurses, and physicians to operate the equipment and follow stroke protocols. After the initial investment in software, implementation, testing, and staff training, the system has been maintained since November 2021 without requiring additional financial or personnel resources at either the DMC or CSC.

An ongoing implementation study aims to evaluate the effectiveness of our approach in decreasing onset-to-treatment time and enhancing the speed of stroke treatment. Future studies should further assess cost-effectiveness. Overall, this approach offers a practical and scalable solution to improve stroke care in regions with limited access to specialized treatment.

**Table 1:**
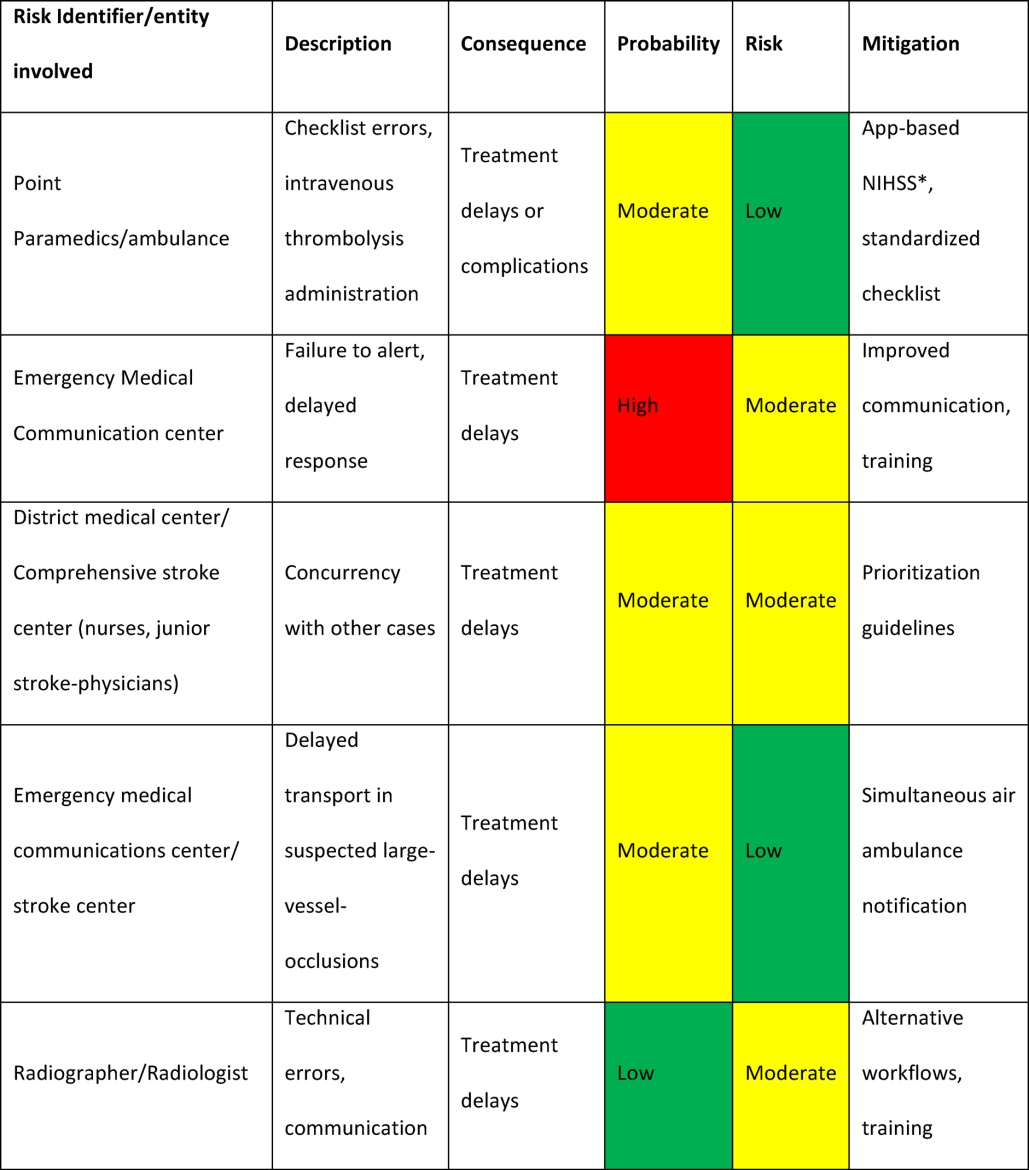
Simplified risk assessment table with proposals for mitigation.

*NIHSS: The National Institute of Health Stroke Scale.

## Conclusion

This study demonstrates the feasibility of using a remotely controlled CT system at a DMC to improve the timely assessment and treatment of AIS in a rural setting. By integrating this technology with real-time communication with stroke physicians at a CSC and on-site paramedic assessments, we have established an effective model for administering IVT in a remote setting, addressing the challenges of delivering timely acute ischemic stroke care in rural regions.

## Data Availability

The datasets analysed during the current study is available from the corresponding author on reasonable request.

## Abbreviations

AE: Adverse events
AIS: Acute ischemic stroke
CT: Computer tomography
CSC: Comprehensive Stroke Center
DICOM: Digital imaging and communications in medicine
DMC: District Medical Center
DNT: Door-to-needle time
EMC: Emergency Medical Communication Center
GDPR: General data protection regulation
ISO: International Organization for Standardization
IVT: Intravenous thrombolysis
KVM: Keyboard, video, mouse
LVO: Large vessel occlusion
MRI: Magnetic resonance imaging
MSU: Mobile stroke units
NIHSS: The National Institute of Health Stroke Scale
OTT: Onset-to-treatment time
REC: Regional Ethics Committee
SOP: Standard operating procedure
UNN Tromsø: University Hospital of North Norway, Tromsø

## Declarations

### Patient and public statement

A representative from the national patient association for stroke, LHL Stroke and Aphasia, was involved in all phases of the study.
